# Association between alactic base excess on mortality in sepsis patients: a retrospective observational study

**DOI:** 10.1186/s40560-025-00789-9

**Published:** 2025-04-11

**Authors:** Jiahui Liu, Yang Chen, Bin Yang, Jiabao Zhao, Qiang Tong, Yuan Yuan, Ye Kang, Tianshu Ren

**Affiliations:** 1 Department of Pharmacy, General Hospital of Northern Theater Command, No. 83 Wenhua Road, Shenhe District, Shenyang, 110000 Liaoning Province People’s Republic of China; 2 Liverpool Centre for Cardiovascular Science at University of Liverpool, Liverpool John Moores University and Liverpool Heart and Chest Hospital, Liverpool, UK; 3 Department of Cardiovascular and Metabolic Medicine, Institute of Life Course and Medical Sciences, University of Liverpool, Liverpool, UK; 4 The Second Affiliated Hospital of Shenyang Medical College, Heping District, Shenyang, People’s Republic of China

**Keywords:** Alactic base excess, Base excess, Lactate, Sepsis, Intensive care unit, Mortality

## Abstract

**Background:**

Sepsis is a life-threatening condition often associated with metabolic and acid–base imbalances. Alactic base excess (ABE) has emerged as a novel biomarker to assess metabolic disturbances in critically ill sepsis patients, but its prognostic value remains underexplored. We aimed to investigate the relationship between ABE and 30-day/90-day ICU all-cause mortality in a large sepsis cohort in the intensive care unit (ICU) setting.

**Methods:**

This study utilised data from a large US ICU sepsis cohort. ABE was calculated as the sum of lactate and base excess (BE) values from the first day of ICU admission. Patients were divided into quartiles based on ABE values. Kaplan–Meier survival analysis, Cox proportional hazards models, and restricted cubic spline analyses were used to examine the associations between ABE and mortality outcomes. The predictive performance of ABE combined with the SOFA score was assessed using the area under the curve, Net Reclassification Improvement, and Integrated Discrimination Improvement.

**Results:**

17,099 patients with sepsis were included in this analysis, with median (IQR) age of 67.82 (56.80, 78.04) years and 59.7% males. Our analysis revealed a *U*-shaped association between ABE and 30-day and 90-day ICU all-cause mortality. Both the lowest (Q1) and highest (Q4) quartiles of ABE were linked to increased mortality risks, with 30-day mortality showing HRs of 1.27 (95% CI 1.13–1.44) for Q1 and 1.17 (95% CI 1.06–1.31) for Q4, while 90-day mortality showed HRs of 1.28 (95% CI 1.16–1.44) for Q1, 1.12 (95% CI 1.02–1.23) for Q2, and 1.22 (95% CI 1.11–1.34) for Q4. ABE demonstrated superior predictive performance for mortality compared to BE and lactate. Incorporating ABE into the SOFA score improved predictive performance, emphasizing ABE’s value in better risk stratification. The identified thresholds (2.5 mmol/L for 30-day mortality and 2.2 mmol/L for 90-day mortality) indicate optimal ABE levels that may be associated with improved survival outcomes.

**Conclusions:**

ABE demonstrated a *U*-shaped association with 30-day and 90-day ICU all-cause mortality in critically ill sepsis patients, suggesting its superiority over BE and lactate as a predictive biomarker. Incorporating ABE with the SOFA score may further enhance prognostic predictions. Further studies are needed to determine whether ABE should serve solely as a biomarker for monitoring the clinical course or could also be considered a potential therapeutic target.

**Supplementary Information:**

The online version contains supplementary material available at 10.1186/s40560-025-00789-9.

## Introduction

Sepsis is defined as life-threatening organ dysfunction caused by a dysregulated host response to infection [1]. Sepsis is one of the most common and critical conditions in the intensive care unit (ICU) and is a leading cause of mortality among ICU patients. Sepsis can rapidly progress to septic shock, which has a very high mortality rate. Statistics indicate that the mortality rate for sepsis can reach 25–30%, while the mortality rate for septic shock is even higher, potentially exceeding 40% [1, 2]. Early identification and timely intervention are crucial for improving the prognosis of sepsis patients. Studies have shown that administering antibiotics and fluid resuscitation rapidly within the “golden hour” of sepsis management can significantly reduce mortality [3, 4]. Alactic base excess (ABE) is a parameter used to assess acid–base balance in vivo and allows for a better determination of the source of lactate (e.g., whether or not renal function is impaired) as compared to lactate values alone, thus helping to accurately assess whether tissue perfusion is being effectively improved and guiding fluid resuscitation strategies [5]. ABE is calculated by adjusting the traditional base excess (BE) to exclude the impact of lactate-induced metabolic acidosis, helping to distinguish metabolic acidosis caused by non-lactate factors, such as chloride imbalance or renal dysfunction [6, 7].

Lactate is widely recognized as a key biomarker for assessing tissue hypoxia and metabolic stress in sepsis, and elevated lactate levels have been strongly associated with increased mortality risk [8, 9]. Similarly, BE is commonly used to evaluate acid–base balance and buffering capacity, with abnormal BE levels linked to worse outcomes in critically ill patients [10]. However, lactate may be influenced by factors beyond tissue hypoxia, such as hypermetabolism or impaired clearance. BE, on the other hand, does not differentiate lactate-driven acidosis from other metabolic imbalances. ABE addresses these gaps by isolating non-lactate metabolic disturbances, providing a more comprehensive assessment of acid–base status.

Changes in ABE are typically associated with metabolic acidosis, and existing studies have shown that ABE is a risk factor for short-term mortality in shock patients [11]. It is also closely related to the prognosis of patients with myocardial infarction and heart failure [12]. Joaquín Cantos and colleagues found that in sepsis and septic shock patients without kidney injury, a negative ABE (< − 3 mmol/L) is a strong predictor of in-hospital mortality [13]. However, studies focus on ICU sepsis patients are still limited. Therefore, the actual predictive value of ABE as a prognostic indicator still requires further validation. Physiologically, ABE is primarily influenced by renal tubular reabsorption, tubular ammonia production, bicarbonate buffering capacity, and non-lactate metabolites (e.g., ketoacids and uremic toxins), making it a more sensitive indicator of metabolic abnormality in the presence of renal insufficiency, compensated respiratory acidosis, and chloride imbalance [14, 15]. In addition, in shock resuscitation, mechanical ventilation management, and early sepsis, ABE can help to differentiate between lactic and non-lactic sources of acidosis, providing an important basis for optimising fluid management, adjusting ventilation strategies, and assessing organ perfusion.

Therefore, this study aimed to investigate the relationships between ABE with 30-day and 90-day all-cause mortality outcomes of sepsis patients in the ICU setting.

## Methods

### Data resource

The data for this study were extracted from the Medical Information Mart for Intensive Care IV (MIMIC-IV) version 2.0, which is a publicly accessible anonymised digital health record database of around 76,000 ICU admissions from 2008 to 2019 at Beth Israel Deaconess Medical Center in Boston, Massachusetts, US [16]. The ethical approval of MIMIC IV was approved by the institutional review boards of the BIDMC (2001-P-001699/14) and Massachusetts Institute of Technology (No. 0403000206). One of the authors (Yang Chen) has completed the Human Subjects Research Training Certificate for accessing MIMIC IV (Certificate No. 53753450) and has relevant experience working with the MIMIC IV [17, 18]. All procedures involving human participants in this study adhered to the ethical standards of the institutional and national research committees, in line with the 1964 Declaration of Helsinki and its subsequent amendments.

### Study population

The sepsis patients were identified according to the Third International Consensus Definitions for Sepsis and Septic Shock criteria, including: (*i*) patients with suspected infection defined as the presence of a bloodstream pathogen or the administration of antibiotics and (*ii*) patients with SOFA scores ≥ 2 [19]. Exclusion criteria of this study were as follows: (*i*) patient underwent kidney transplant; (*ii*) patient received renal replacement therapy; (*iii*) patients aged < 18 years; (*iv*) patients without values of lactate and base excess at first day after admission; (*v*) patients whose length of ICU stay < 24 h; (*vi*) records of multiple hospital or ICU admission; and (*vii*) patients without records of intake and output at first day after admission.

### Definition of ABE

In this study, ABE was calculated using the formula: ABE (mmol/L) = BE (mmol/L) + lactate (mmol/L). Here, both BE and lactate values were derived from the first arterial blood gas analysis performed at the time of admission. This timing is chosen, because initial ABE reflects the patient’s baseline acid–base status upon ICU entry, and it captures the severity of metabolic acidosis and tissue hypoxia at the most critical timepoint, allowing for timely risk stratification and intervention. In addition, both the BE and lactate values are measured in millimoles per liter.

### Study outcomes

Our primary outcomes were 30-day and 90-day all-cause mortality outcomes. Our secondary outcomes were ICU mortality, hospital mortality, ICU length of stay, and hospital length of stay, which were analysed descriptively only.

### Covirates extraction

We extracted the following covariates: demographics variables (e.g., age and sex), vital signs (e.g., heart rate and systolic blood pressure), SOFA, comorbidities (e.g., coronary artery disease and myocardial infarction), laboratory results (e.g., calcium and potassium), medications (e.g., vasopressors and corticosteroids), and receiving mechanical ventilation at first day after admission. Laboratory tests were analyzed using only the first value recorded on the first day after admission.

### Statistical analysis

The covariates in this study had different proportions of missing values, as shown in Supplementary Table S1, with the highest proportion reaching about 15%, which were then imputated. We applied multiple imputations using the ‘miceforest’ package in Python, performing 20 iterations (n = 20) to ensure stable and reliable imputations. Continuous variables were summarized using either the mean with standard deviation (SD) or the median with interquartile range (IQR), depending on the normality of the data. Then, we applied analysis of variance or the Kruskal–Wallis test, as suited to the distribution of the data. Categorical variables were presented as counts and percentages, with group comparisons made using the Chi-squared test or Fisher’s exact test, depending on the expected frequency counts.

We divided the cohort into four groups based on the 25th, 50th and 75th values of ABE: Quartile [Q1] (ABE < − 1.40 mmol/L), Q2 (− 1.40 ≤ ABE < 1.20 mmol/L), Q3 (1.20 ≤ ABE < 3.20 mmol/L), and Q4 (ABE ≥ 3.20 mmol/L). Survival probabilities were estimated using the Kaplan–Meier method, and differences among groups were assessed using the log-rank test. Subsequently, we applied the Cox proportional hazards models to assess the associations between ABE with 30-day and 90-day all-cause mortality outcomes in sepsis patients, and calculated hazard ratios (HR) and 95% confidence intervals (95% CI). We then adjusted four different models, including Model I: no adjustment; Model II: adjustment by age, sex, body mass index and race; Model III: based on Model II and further adjusted by comorbidities (acute respiratory failure, sepsis-associated encephalopathy, sepsis shock, acute kidney injury, myocardial infarction, congestive heart failure, peripheral vascular disease, coronary artery disease, cerebrovascular disease, chronic pulmonary disease, hepatic disease, malignant cancer, and diabetes mellitus). Model IV: based on Model III and further adjusted by vital signs (heart rate, systolic blood pressure, diastolic blood pressure, oxygen saturation, respiratory rate), SOFA, laboratory results (glucose, anion gap, bicarbonate, blood urea nitrogen, calcium, chloride, sodium, potassium, phosphorus, creatinine, estimated glomerular filtration rate, partial pressure of carbon dioxide, potential of hydrogen), medications (vasopressors, antibiotic drugs, furosemide, corticosteroids), interventions (renal replacement therapy, mechanical ventilation), fluid input, and fluid output. The variables included in the model were tested using Pearson correlation and variance inflation factor analysis. All correlation coefficients were less than 0.8, and variance inflation factor values were below 8, indicating no significant multicollinearity (Supplementary Figures S1, S2). In addition, restricted cubic spline (RCS) analyses adjusted by Model IV were used to assess the relationships between ABE with 30-day and 90-day all-cause mortality outcomes. If a non-linearity relationship was detected, a recursive algorithm was applied to identify an optimal inflection point, after which a two-segment Cox proportional hazards regression was performed on either side of the identified breakpoint to evaluate the differing hazard ratios. Furthermore, to explore potential effect modifiers in the relationship between ABE and mortality, we performed predefined subgroup analyses based on age, sex, obesity, and AKI severity. These factors were selected due to their biological relevance and potential impact on acid–base balance and clinical outcomes. Older adults have a diminished physiological reserve and reduced buffering capacity, which may impair their ability to compensate for metabolic derangements, making ABE dynamics particularly relevant in this population. Sex-based differences in hormonal and metabolic regulation could influence acid–base homeostasis, lactate metabolism, and renal bicarbonate handling, potentially modifying ABE’s predictive value. Obesity is associated with altered metabolic and respiratory physiology, including increased carbon dioxide production, impaired ventilatory compensation, and higher baseline lactate levels, all of which may affect the interpretation of ABE in sepsis. Given the kidney’s central role in acid–base balance through bicarbonate reabsorption and hydrogen ion excretion, AKI severity directly impacts metabolic acidosis and lactate clearance, making it a critical factor in evaluating ABE’s prognostic utility. By assessing these subgroup analyses, we aimed to determine whether ABE’s prognostic value varies across different physiological conditions, further assessing its role in risk stratification among critically ill sepsis patients.

To evaluate the standalone predictive performance of ABE, BE, and lactate for 30-day and 90-day all-cause mortality in sepsis patients, we calculated the area under the receiver operating characteristic curve (AUC) for each biomarker individually. Pairwise comparisons of the AUC values were performed using the DeLong test to statistically assess whether ABE outperformed BE and lactate. In addition, to evaluate the predictive performance of ABE in combination with current ICU severity score for 30-day and 90-day all-cause mortality in sepsis patients, we incorporated ABE into SOFA. The combined model (ABE + SOFA) was compared against the baseline model (SOFA score alone) to assess improvements in discrimination and reclassification. We calculated the AUC for both models and employed the DeLong test to statistically compare the AUC values. In addition, we utilized the Net Reclassification Improvement (NRI) and Integrated Discrimination Improvement (IDI) indices to further quantify the incremental prognostic value of including ABE.

All analyses were applied utilising SPSS Statistics (version 27, USA), Python (version 3.11.1, USA) and R (version 4.3.2, Austria). A two-tailed *P* < 0.05 was interpreted as statistically significant.

## Results

### Baseline characteristics

**Fig. 1:**
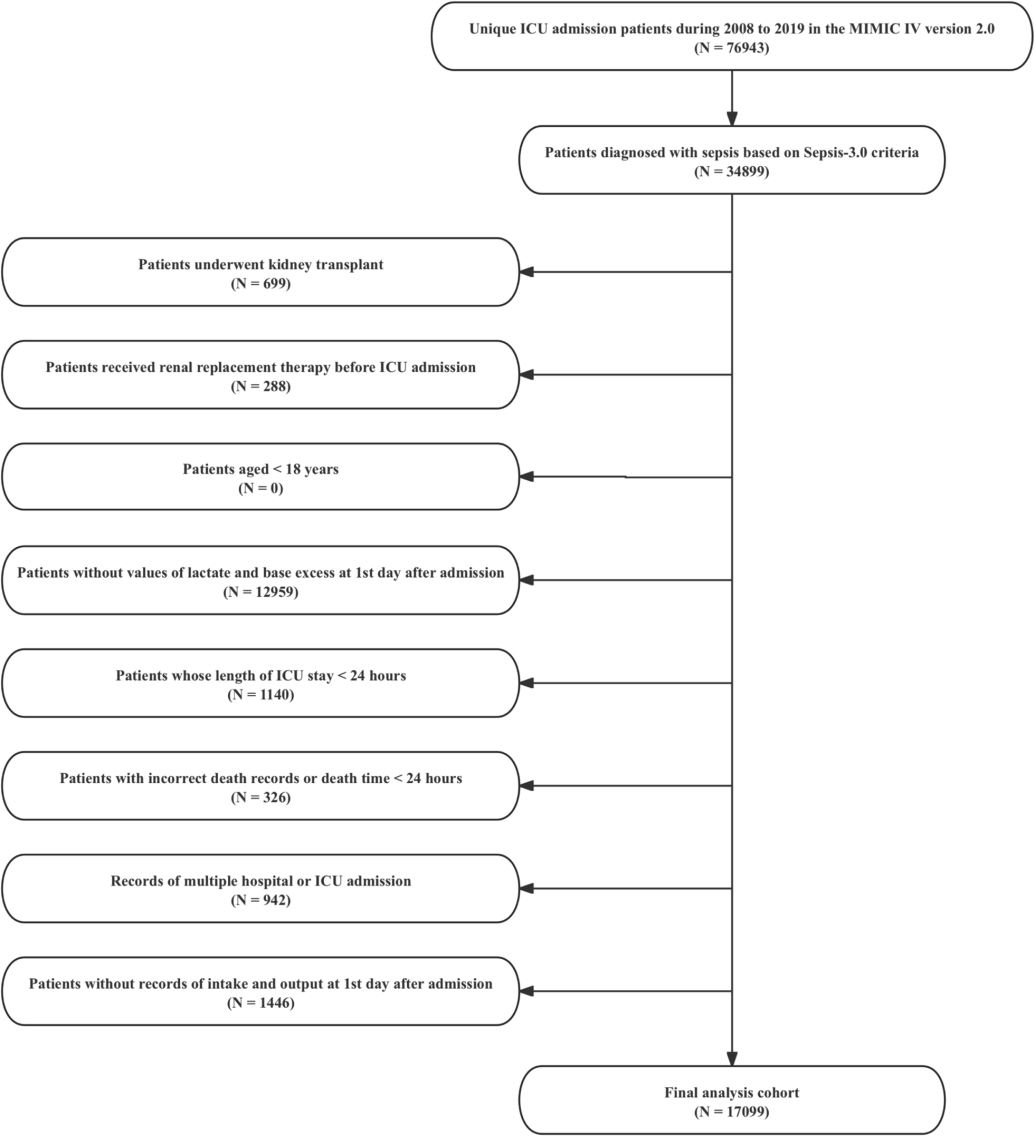
Flowchart of this study. ICU, intensive care unit; MIMIC, Medical Information Mart for Intensive Care

We ultimately included 17,099 patients with sepsis in this study (Fig. 1), with a median (IQR) age of 67.82 (56.80, 78.04) years and 59.7% males (Table 1). Overall, there were significant differences in age, gender and ethnicity among the different ABE subgroups. In addition, heart rate, systolic blood pressure and oxygen saturation were significantly different among the four quartile groups (*P* < 0.001). In terms of comorbidities, acute kidney injury, congestive heart failure, cerebrovascular events and hepatic disease were more common in the high ABE group (Q3, Q4), while myocardial infarction and malignant cancer were more common in Q1 and Q2. Laboratory results also showed that as the ABE quartiles increased, the anion gap, glucose and bicarbonate levels increased significantly (*P* < 0.001).

**Table 1:** Baseline characteristics of sepsis patients stratified by ABE quartiles

	Overall (*N* = 17,099)	Q1 (*N* = 4270)	Q2 (*N* = 4018)	Q3 (*N* = 4518)	Q4 (*N* = 4292)	*P*
Age, years	67.82 (56.80, 78.04)	66.76 (54.93, 77.99)	68.13 (56.94, 77.94)	67.42 (57.11, 77.14)	68.96 (58.17, 79.07)	0.003
Male, %	10,210 (59.7)	2230 (54.6)	2460 (61.2)	2951 (65.3)	2469 (57.5)	< 0.001
White, %	11,449 (67.0)	2742 (64.2)	2696 (67.1)	3131 (69.3)	2880 (67.1)	< 0.001
Body mass index, kg/m^2^	27.79 (24.14, 32.46)	27.49 (23.87, 32.11)	27.76 (24.24, 32.22)	28.00 (24.39, 32.28)	28.00 (24.03, 33.08)	0.139
Vital signs						
Heart rate, bpm	87 (77, 103)	93 (80, 109)	87 (76, 101)	85 (76, 98)	87 (77, 102)	< 0.001
SBP, mmHg	117 (103, 135)	115 (101, 133)	117 (103, 134)	117 (104, 134)	120 (106, 137)	< 0.001
DBP, mmHg	64 (54, 75)	63 (53, 75)	63 (53, 75)	64 (55, 75)	65 (55, 76)	0.207
Respiratory rate, bpm	18 (15, 23)	20 (16, 24)	18 (15, 22)	17 (14, 22)	18 (15, 23)	< 0.001
Oxygen saturation, %	99 (96, 100)	98 (95, 100)	99 (96, 100)	99 (96, 100)	99 (96, 100)	< 0.001
SOFA	3.00 (2.00, 5.00)	4.00 (2.00, 6.00)	3.00 (2.00, 5.00)	3.00 (2.00, 4.00)	3.00 (2.00, 4.00)	< 0.001
Comorbidities, *n* (%)						
ARFA	5965 (34.9)	1837 (43.0)	1340 (33.3)	1295 (28.7)	1493 (34.8)	< 0.001
SAE	12,903 (75.5)	3378 (79.1)	2949 (73.4)	3249 (71.9)	3327 (77.5)	< 0.001
Sepsis shock	3226 (18.9)	1451 (34.0)	691 (17.2)	548 (12.1)	536 (12.5)	< 0.001
Acute kidney injury						< 0.001
Stage I	2256 (13.2)	409 (9.6)	550 (13.7)	698 (15.4)	599 (14.0)	
Stage II	5171 (30.2)	1228 (28.8)	1249 (31.1)	1380 (30.5)	1314 (30.6)	
Stage III	2008 (11.7)	870 (20.4)	404 (10.1)	334 (7.4)	400 (9.3)	
Myocardial infarction	3328 (19.5)	899 (31.1)	808 (20.1)	881 (19.5)	740 (17.2)	< 0.001
CHF	5284 (30.9)	1285 (30.1)	1153 (28.7)	1271 (28.1)	1575 (36.7)	< 0.001
PVD	2246 (13.1)	568 (13.3)	551 (13.7)	635 (14.1)	492 (13.1)	0.002
CVA	2476 (14.5)	530 (12.4)	605 (15.1)	722 (16.0)	619 (14.4)	< 0.001
CPD	4718 (27.6)	1101 (25.8)	1076 (26.8)	1143 (25.3)	1398 (32.6)	< 0.001
Hepatic disease	2611 (15.3)	901 (21.1)	562 (14.0)	551 (12.2)	597 (13.9)	< 0.001
Malignant cancer	2480 (14.5)	684 (16.0)	596 (14.8)	574 (12.7)	626 (14.6)	< 0.001
Diabetes mellitus	5262 (30.8)	1345 (31.5)	1172 (29.2)	1347 (29.8)	1398 (32.6)	0.002
Laboratory results						
ABE, mmol/L	1.20 (− 1.40, 3.20)	− 4.00 (− 6.40, − 2.60)	0.20 (− 0.60, 0.80)	2.00 (1.60, 2.50)	5.10 (4.00, 7.20)	< 0.001
BE, mmol/L	− 1.00 (− 4.00, 1.00)	− 7.00 (− 10.00, − 5.00)	− 2.00 (− 3.00, − 1.00)	0.00 (0.00, 1.00)	3.00 (2.00, 5.00)	< 0.001
Lactate, mmol/L	1.80 (1.20, 2.80)	2.10 (1.30, 3.50)	1.70 (1.10, 2.70)	1.80 (1.40, 2.50)	1.80 (1.20, 2.80)	< 0.001
Anion gap, mmol/L	14.00 (12.00, 17.00)	16.00 (13.00, 20.00)	14.00 (11.00, 17.00)	13.00 (11.00, 16.00)	13.00 (11.00, 16.00)	< 0.001
Glucose, mg/dL	137.00 (111.00, 173.00)	140.00 (110.00, 187.00)	136.00 (111.00, 170.00)	135.00 (112.00, 167.00)	135.00 (111.00, 171.00)	< 0.001
Bicarbonate, mmol/L	22.00 (20.00, 25.00)	18.00 (16.00, 20.00)	22.00 (20.00, 24.00)	23.00 (22.00, 25.00)	26.00 (23.00, 29.00)	< 0.001
BUN, mg/dL	20.00 (14.00, 33.00)	27.00 (17.00, 48.00)	20.00 (14.00, 30.00)	17.00 (13.00, 26.00)	19.00 (13.00, 30.00)	< 0.001
Calcium, mg/dL	8.10 (7.70, 8.60)	7.80 (7.30, 8.40)	8.10 (7.70, 8.60)	8.20 (7.80, 8.60)	8.40 (7.90, 8.80)	< 0.001
Chloride, mmol/L	106.00 (101.00, 110.00)	107.00 (103.00, 111.00)	107.00 (102.00, 110.00)	106.00 (102.00, 110.00)	103.00 (98.00, 108.00)	< 0.001
Sodium, mmol/L	139.00 (136.00, 141.00)	138.00 (125.00, 141.00)	139.00 (136.00, 141.00)	139.00 (136.00, 141.00)	139.00 (137.00, 142.00)	0.001
Potassium, mmol/L	4.20 (3.80, 4.60)	4.30 (3.80, 4.90)	4.20 (3.80, 4.60)	4.10 (3.80, 4.50)	4.00 (3.60, 4.50)	< 0.001
Phosphorus, mg/dL	3.50 (2.90, 4.40)	4.00 (3.10, 5.20)	3.50 (2.90, 4.40)	3.40 (2.80, 4.10)	3.40 (2.80. 4.10)	< 0.001
eGFR, mL/min/1.73m^2^	72.77 (43.25, 102.20)	46.58 (26.11, 77.67)	73.09 (46.13, 101.46)	83.85 (57.92, 106.55)	81.96 (53.38, 110.10)	< 0.001
PaCO_2_, %	41.00 (35.00, 47.00)	39.00 (33.00, 46.00)	41.00 (35.00, 46.00)	41.00 (36.00, 46.00)	42.00 (37.00, 50.00)	< 0.001
Potential of hydrogen	7.31 (7.38, 7.43)	7.29 (7.21, 7.34)	7.36 (7.32 7.41)	7.40 (7.36, 7.44)	7.42 (7.38, 7.47)	< 0.001
Interventions, %						
RRT	691 (4.0)	335 (7.8)	115 (2.9)	99 (2.2)	142 (3.3)	< 0.001
Mechanical ventilation	10,639 (62.2)	2749 (64.4)	2509 (62.4)	2740 (25.8)	2641 (61.5)	0.003
Medications, %						
Vasopressors	9605 (56.2)	2725 (63.8)	2276 (56.6)	2506 (55.5)	2098 (48.9)	< 0.001
Corticosteroids	1185 (6.9)	411 (9.6)	268 (6.7)	239 (5.3)	267 (6.2)	< 0.001
Antitotic drugs	14,950 (87.4)	3756 (88.0)	3500 (87.1)	3979 (88.1)	3715 (86.5)	0.099
Furosemide	5399 (31.6)	927 (21.7)	1186 (29.5)	1637 (36.2)	1649 (38.4)	< 0.001
Fluid output, *L*	2.34 (1.39, 3.48)	1.96 (1.03, 3.23)	2.40 (1.49, 3.50)	2.55 (1.62, 3.60)	2.37 (1.47, 3.49)	< 0.001
Fluid input, *L*	1.88 (9.25, 3.09)	2.37 (1.22, 3.90)	1.95 (1.00, 3.06)	1.74 (0.87, 2.77)	1.60 (0.73, 2.61)	< 0.001

Q1 (ABE < − 1.40), Q2 (− 1.40 ≤ ABE < 1.20), Q3 (1.20 ≤ ABE < 3.20), and Q4 (ABE ≥ 3.20)

*ABE* alactic base excess; *AFRA* acute respiratory failure; *BUN* blood urea nitrogen; *CHF* congestive heart failure; *CPD* chronic pulmonary disease; *CVA* cerebrovascular disease; *DBP* diastolic blood pressure; *eGFR* estimated glomerular filtration rate; *PaCO*_*2*_ partial pressure of carbon dioxide; *PVD* peripheral vascular disease; *RRT* renal replacement therapy; *SAE* sepsis associated encephalopathy; *SBP* systolic blood pressure; *SOFA* Sequential Organ Failure Assessment

### Study outcomes for ABE quartiles

**Fig. 2:**
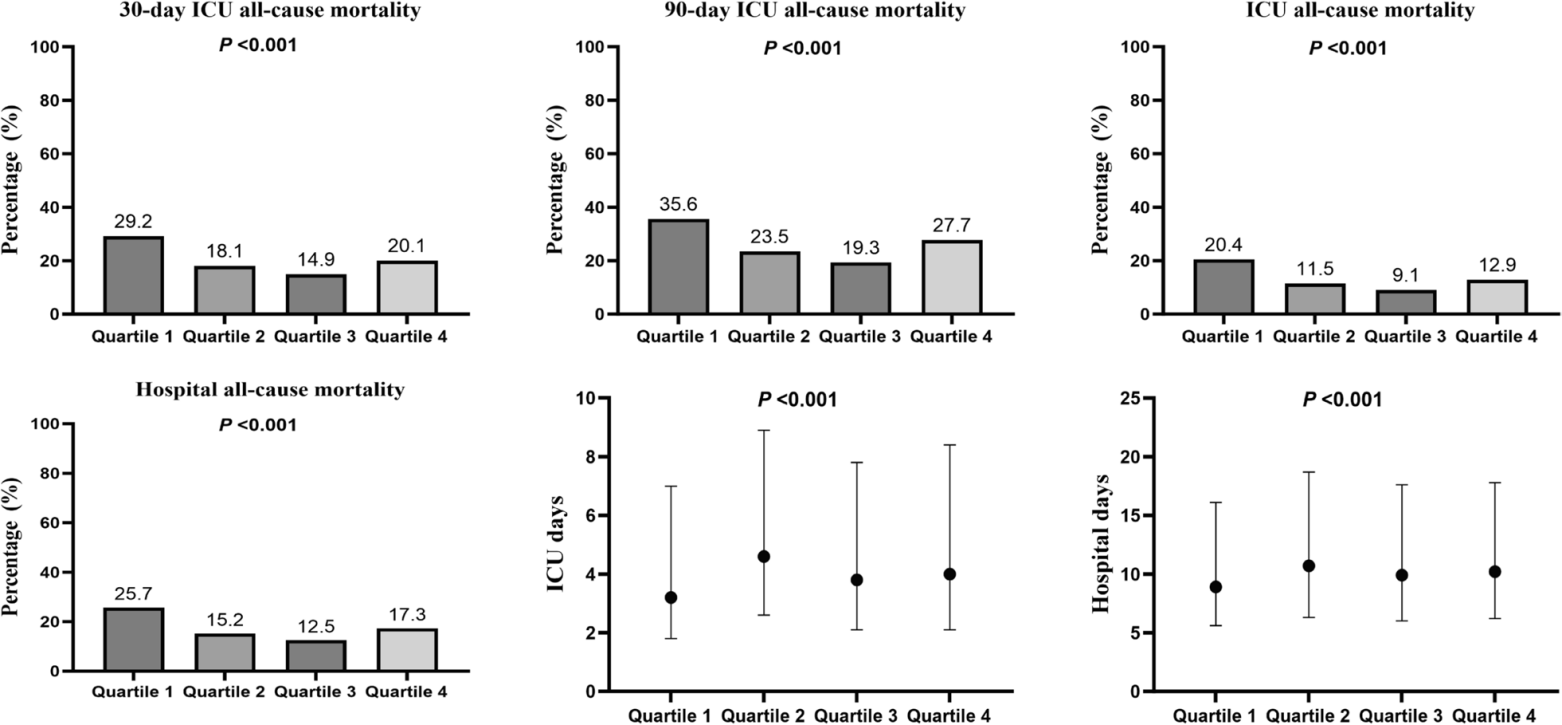
Distribution of clinical outcomes across alactic base excess quartiles in sepsis patients. ICU, intensive care unit

Figure 2 demonstrates a different distribution of adverse clinical outcomes across the ABE quartiles. ICU and hospital all-cause mortality show a *U*-shaped trend, with the lowest and the highest ABE groups having the highest mortality risk (*P* < 0.001). Specifically, for 30-day ICU all-cause mortality, Q1 (29.2%) > Q4 (20.1%) > Q2 (18.1%) > Q3 (14.9%), for 90-day ICU all-cause mortality, Q1 (35.6%) > Q4 (27.7%) > Q2 (23.5%) > Q3 (19.3%). Similarly, for ICU and hospital all-cause mortality, the ABE quartiles followed a similar pattern. There were significant differences in ICU and hospital length of stay across different ABE quartiles, especially the Q1, which had a significantly shorter length of stay (*P* < 0.001).

### Kaplan–Meier survival curves for 30-day and 90-day mortality by ABE quartiles

**Fig. 3:**
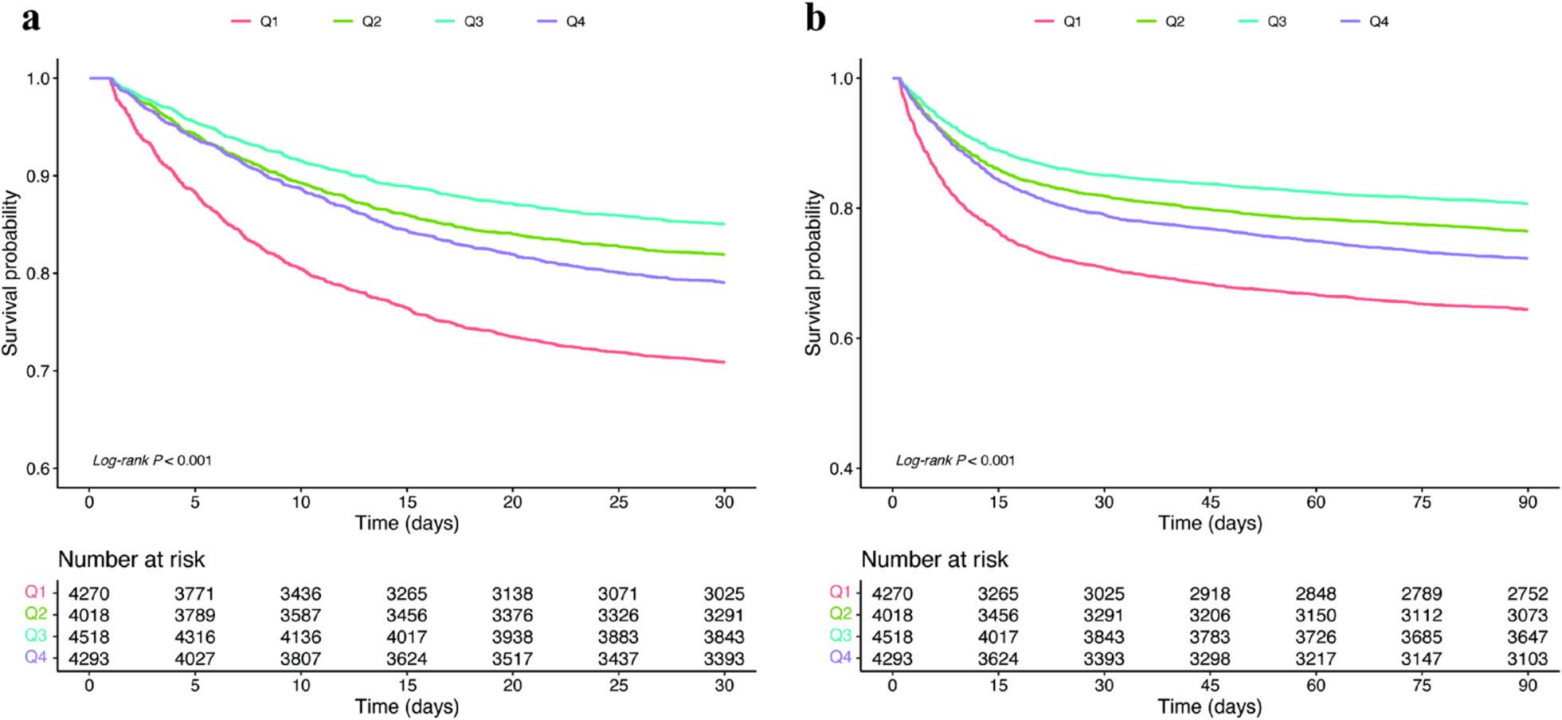
Kaplan–Meier survival curves for sepsis patients stratified by ABE quartiles. **a** 30-day all-cause mortality [*log-rank P* < 0.001]; **b** 90-day all-cause mortality [*log-rank P* < 0.001]. ABE, alactic base excess

The Kaplan–Meier survival analysis demonstrates a clear stratification of survival outcomes based on ABE quartiles (Fig. 3). Patients in the lowest ABE quartile (Q1) consistently showed the worst survival rates at both 30 and 90 days compared to those in higher quartiles (Q2, Q3, and Q4). The log-rank test confirmed significant differences in survival probabilities across groups (both *log-rank P* < 0.001).

### Associations of ABE with 30-day and 90-day ICU all-cause mortality

From Table 2, after adjusting for Model IV, the multivariate Cox proportional hazards model showed that Q1 and Q4 of ABE were associated with a higher risk of 30-day ICU all-cause mortality compared to Q3 of ABE (Q1: HR = 1.27, 95% CI 1.13–1.44; Q4: HR = 1.17, 95% CI 1.06–1.31). Moreover, Q1, Q2 and Q4 were associated with a higher risk of 90-day ICU all-cause mortality compared to Q3 of ABE (Q1: HR = 1.28, 95% CI 1.16–1.44; Q2: HR 1.12, 95% CI 1.02–1.23; Q4: HR 1.22, 95% CI 1.11–1.34).

**Table 2:** Associations between ABE with 30-day and 90-day ICU all-cause mortality

		Model I		Model II		Model III		Model IV	
		HR (95% CI)	*P*	HR (95% CI)	*P*	HR (95% CI)	*P*	HR (95% CI)	*P*
30-day ICU all-cause mortality*									
	Q1	2.17 (1.97–2.38)	< 0.001	2.14 (1.95–2.35)	< 0.001	1.43 (1.30–1.58)	< 0.001	1.27 (1.13–1.44)	< 0.001
	Q2	1.24 (1.11–1.37)	< 0.001	1.22 (1.10–1.35)	< 0.001	1.08 (0.98–1.20)	0.133	1.09 (0.98–1.21)	0.129
	Q3	*Reference*		*Reference*		*Reference*		*Reference*	
	Q4	1.45 (1.31–1.60)	< 0.001	1.39 (1.25–1.53)	< 0.001	1.31 (1.19–1.45)	< 0.001	1.17 (1.06–1.31)	0.003
90-day ICU all-cause mortality*									< 0.001
	Q1	2.01 (1.93–2.28)	< 0.001	2.09 (1.92–2.27)	< 0.001	1.42 (1.31–1.55)	0.029	1.28 (1.16–1.44)	< 0.001
	Q2	1.25 (1.14–1.37)	< 0.001	1.24 (1.13–1.36)	< 0.001	1.11 (1.01–1.22)	< 0.001	1.12 (1.02–1.23)	0.020
	Q3	*Reference*		*Reference*		*Reference*		*Reference*	
	Q4	1.50 (1.38–1.64)	< 0.001	1.44 (1.32–1.57)	< 0.001	1.37 (1.25–1.49)	< 0.001	1.22 (1.11–1.34)	< 0.001

Model I: unadjusted;

Model II: adjusted for age, sex, body mass index and race;

Model III: based on Model II further adjusted for acute respiratory failure, sepsis associated encephalopathy, sepsis shock, acute kidney injury, myocardial infarction, congestive heart failure, peripheral vascular disease, coronary artery disease, cerebrovascular disease, hepatic disease, malignant cancer, diabetes mellitus;

Model IV: based on Model III further adjusted for heart rate, systolic blood pressure, diastolic blood pressure, oxygen saturation, respiratory rate, Sequential Organ Failure Assessment, glucose, anion gap, bicarbonate, blood urea nitrogen, calcium, chloride, sodium, potassium, phosphorus, creatinine, estimated glomerular filtration rate, partial pressure of carbon dioxide, potential of hydrogen, renal replacement therapy, mechanical ventilation, vasopressor, antibiotics, furosemide, corticosteroids, fluid input, fluid output

*P for trend < 0.001 for each model corresponding to this outcome

*ABE* alactic base excess; *CI* confidence interval; *HR* hazard ratio; *ICU* intensive care unit

### RCS analysis and threshold of ABE with 30-day and 90-day ICU all-cause mortality

**Fig. 4:**
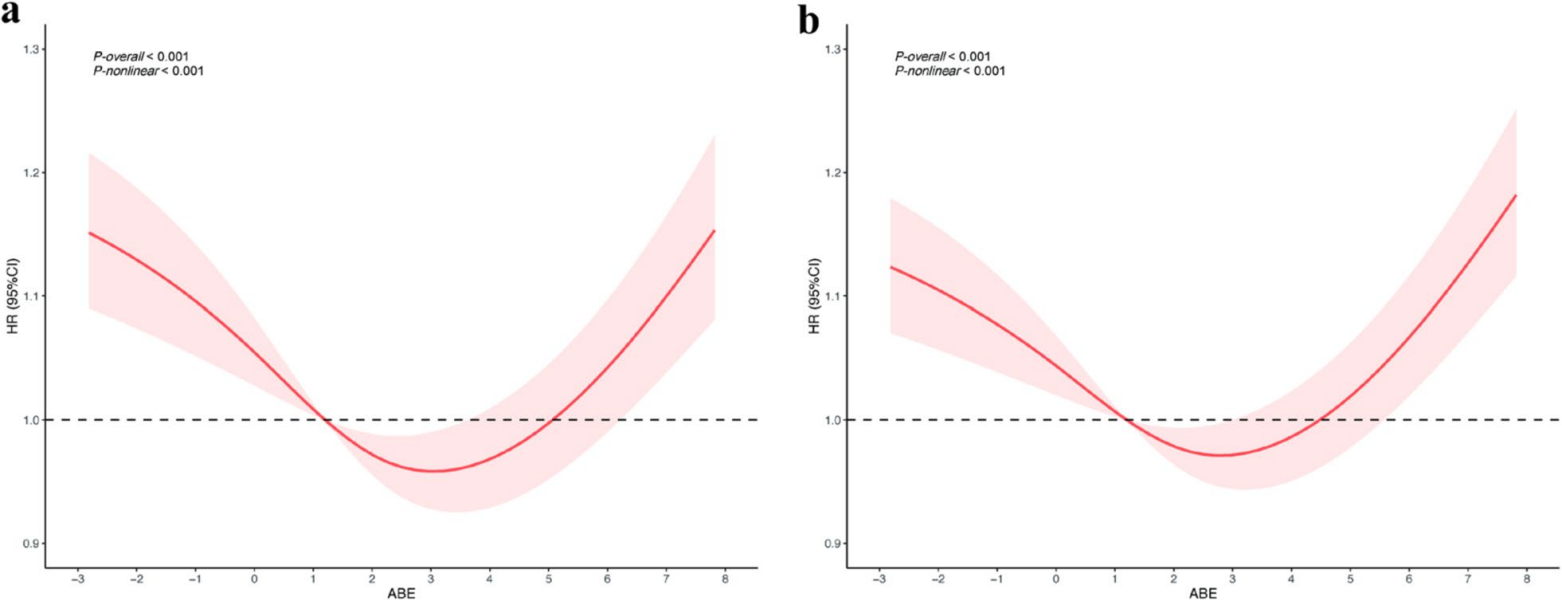
Restricted cubic spline analysis of the relationships between ABE and mortality outcomes in ICU sepsis patients. **a** 30-day all-cause mortality; **b** 90-day all-cause mortality. ABE, alactic base excess; CI, confidence interval; HR, hazard ratio; ICU, intensive care unit

The RCS analysis of Fig. 4 shows a significant nonlinear relationship between ABE and both 30-day and 90-day ICU all-cause mortality (30 days: *P-overall* < 0.001, *P-nonlinear* < 0.001; 90 days: *P-overall* < 0.001, *P-nonlinear* < 0.001), with a clear inflection point between 2.0 and 3.0 mmol/L, suggesting a possible threshold effect. Subsequently, we used a recursive algorithm to determine the inflection points of ABE, which were 2.5 mmol/L for 30-day ICU all-cause mortality and 2.2 mmol/L for 90-day ICU all-cause mortality. Two-stage Cox regression models were performed on both sides of the threshold, and below this threshold, there was a significant negative correlation between ABE and 30-day/90-day all-cause mortality (HR < 1.0, *P* < 0.001). In contrast, above the threshold, ABE was positively associated with an increased risk of 30-day/90-day ICU all-cause mortality (HR > 1.0, *P* < 0.001) (Table 3).

**Table 3:** Threshold effect analysis of ABE on 30-day and 90-day ICU all-cause mortality

Outcomes		effect size (95% confidence interval)	*P*
30-day all-cause mortality	Model 1 Fitting model by standard Cox regression	0.998 (0.992–1.005)	0.668
	Model 2 Fitting model by two-piecewise Cox regression		
	Inflection point = 2.5		
	< 2.5	0.975 (0.966–9.983)	< 0.001
	> 2.5	1.047 (1.033–1.061)	< 0.001
	*P* for likelihood ratio test		< 0.001
30-day all-cause mortality	Model 1 Fitting model by standard Cox regression	1.002 (0.996–1.008)	0.492
	Model 2 Fitting model by two-piecewise Cox regression		
	Inflection point = 2.2		
	< 2.2	0.976 (0.968–9.985)	< 0.001
	> 2.2	1.048 (1.036–1.060)	< 0.001
	*P* for likelihood ratio test		< 0.001

### Subgroup analysis

**Fig. 5:**
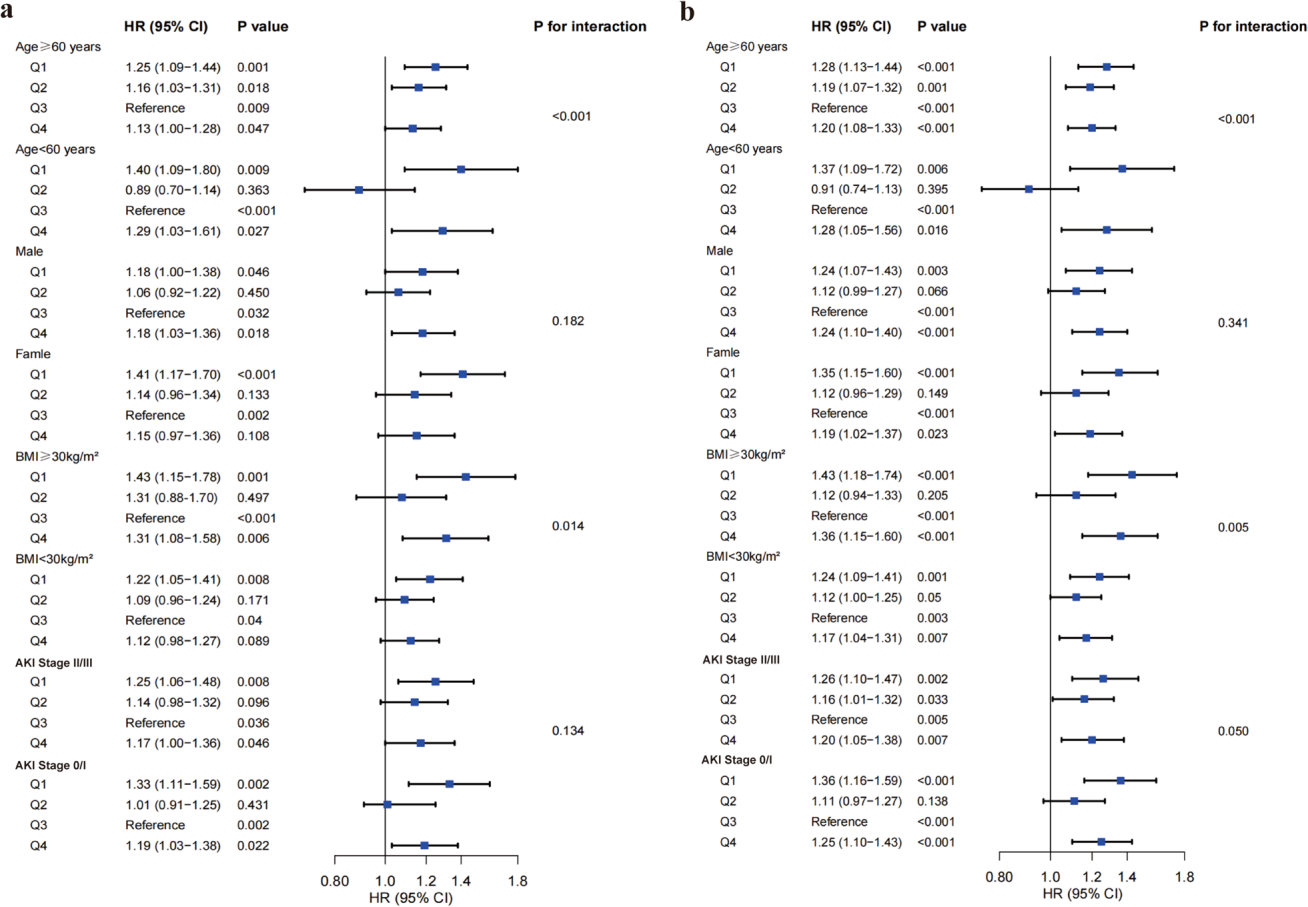
Subgroup analyses for associations between ABE with 30-day (**a**) and 90-day (**c**) ICU all-cause mortality. Q1 (ABE < − 1.40), Q2 (− 1.40 ≤ ABE < 1.20), Q3 (1.20 ≤ ABE < 3.20), and Q4 (ABE ≥ 3.20). ABE, alactic base excess; AKI, acute kidney injury; BMI, body mass index; CI, confidence interval; HR, hazard ratio

Subgroup analysis revealed a significant interaction between age, obesity status and ABE quartile, with Q1 having a significant effect on mortality outcomes (both *P-for-interaction* < 0.05) (Fig. 5). The effect of ABE Q1/Q4 on 30-day/90-day ICU all-cause mortality were particularly pronounced for patients aged ≥ 60 years compared to those under 60 years compared to Q3 of ABE, but overall there were *U*-shaped associations. Similarly, the effect of ABE Q1/Q4 on 30-day/90-day ICU all-cause mortality were more apparent for obesity patients compared to non-obesity patients.

### Predictive performance of ABE for mortality in sepsis patients

For 30-day ICU all-cause mortality, ABE demonstrated a higher AUC (0.580, 95% CI 0.569–0.590) compared to BE (AUC: 0.421, 95% CI 0.410–0.433) and lactate (AUC: 0.444, 95% CI 0.433–0.456), with statistically significant differences (*DeLong test P* < 0.001) (Supplementary Table S2). Similar findings were observed for 90-day ICU all-cause mortality, where ABE achieved an AUC of 0.562 (95% CI 0.552–0.571), again outperforming BE (AUC: 0.435, 95% CI 0.424–0.445) and lactate (AUC: 0.454, 95% CI 0.443–0.464), with all comparisons yielding *DeLong test P* < 0.001 (Supplementary Table S2).

**Fig. 6:**
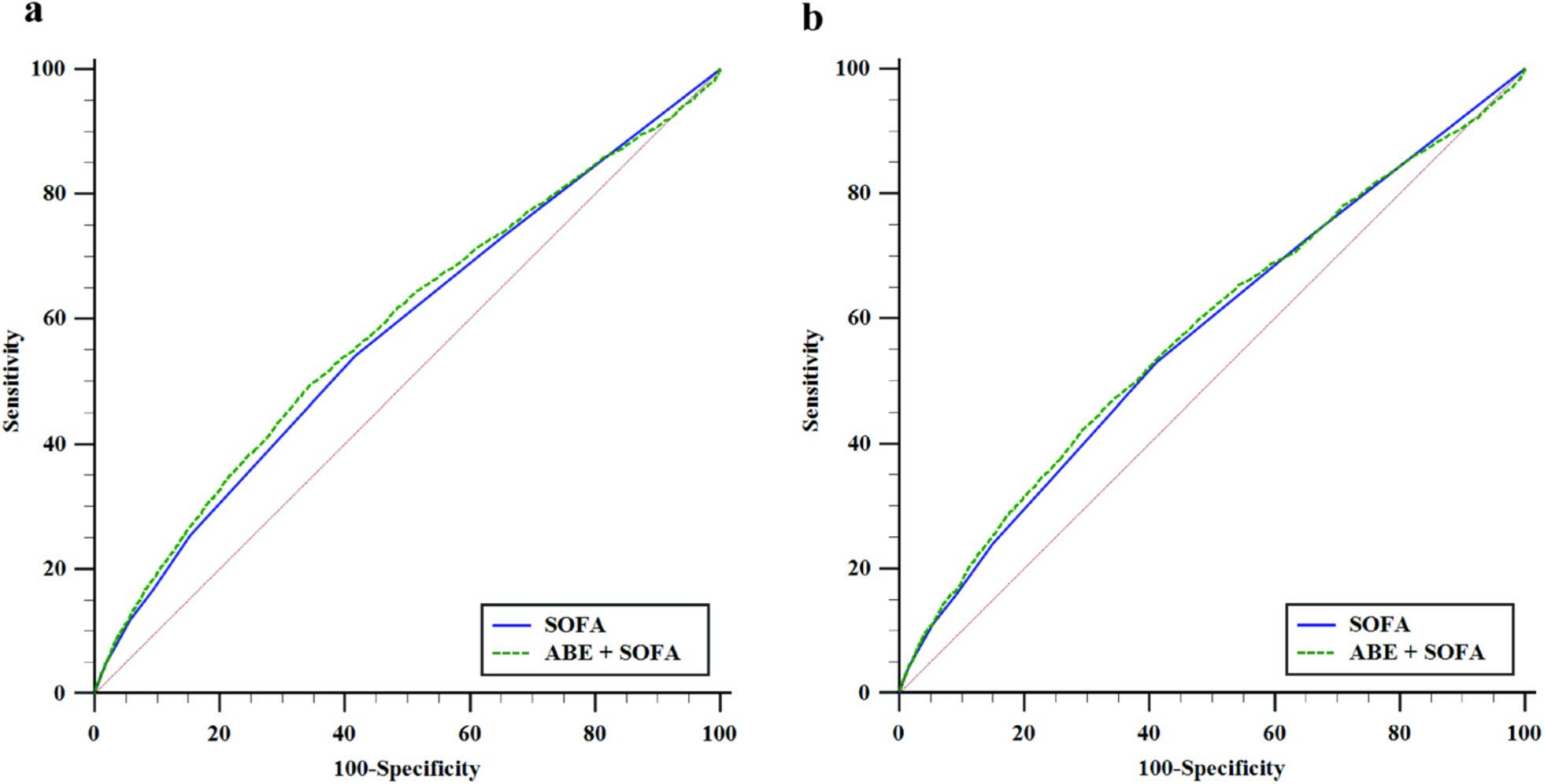
Comparison of receiver operating characteristic curves for mortality prediction using the combined model of ABE and SOFA versus SOFA alone. **a** 30-day ICU all-cause mortality; **b** 90-day ICU all-cause mortality. ABE: alactic base excess; SOFA: sequential organ failure assessment

Incorporating ABE into the SOFA score model significantly improved the predictive performance for both 30-day and 90-day all-cause mortality in sepsis patients (Table 4 and Fig. 6). The AUC for the combined model (ABE + SOFA) was significantly higher than the SOFA model alone (for 30-day ICU all-cause mortality, ABE + SOFA vs. SOFA: 0.59 vs. 0.58, *DeLong test P* < 0.001; for 90-day ICU all-cause mortality, ABE + SOFA vs. SOFA: 0.58 vs. 0.57, *DeLong test P* = 0.017). The combined model also showed substantial improvements in risk reclassification, with an NRI of 16.53% and IDI of 0.13% for 30-day ICU all-cause mortality, and an NRI of 12.58% and IDI of 0.10% for 90-day ICU all-cause mortality, indicating better discrimination and reclassification of high-risk patients when ABE was included.

**Table 4:** Comparison of predictive performance of models incorporating ABE and SOFA for 30-day/90-day all-cause mortality in sepsis patients

	AUC (95% CI)	*DeLong test P*	Continuous NRI (95% CI)	*P*	IDI (95% CI)	*P*
30-day ICU all-cause mortality						
SOFA	0.58 (0.57, 0.59)		*Reference*		*Reference*	
ABE + SOFA	0.59 (0.58, 0.60)	< 0.001	16.53% (12.84%, 20.21%)	< 0.001	0.13% (0.06%, 0.20%)	< 0.001
90-day ICU all-cause mortality						
SOFA	0.57 (0.57, 0.58)		*Reference*		*Reference*	
ABE + SOFA	0.58 (0.57, 0.59)	0.017	12.58% (9.19%, 15.97%)	< 0.001	0.10% (0.03%, 0.14%)	0.004

*ABE* alactic base excess; *AUC* area under curve; *CI* confidence interval; *ICU* intensive care unit; *IDI* Integrated Discrimination Improvement; *NRI* Net Reclassification Improvement; *SOFA* Sequential Organ Failure Assessment

## Discussion

### Main findings

This study uncovered several important findings regarding the relationships between ABE with 30-day and 90-day ICU all-cause mortality outcomes in sepsis patients. First, we observed significant differences across ABE quartiles in baseline characteristics, indicating the clinical heterogeneity of patients based on ABE levels. Second, ABE was associated with a *U*-shaped association with the risk of 30-day and 90-day all-cause mortality in the ICU among patients with sepsis, in which both the lowest (Q1) and highest (Q4) quartiles of ABE were associated with increased 30-day and 90-day ICU and hospital all-cause mortality. This indicates that a deviation from the normal value of ABE may indicate an adverse prognosis. Third, our threshold analysis identified inflection points of ABE (2.5 mmol/L for 30-day ICU all-cause mortality and 2.2 mmol/L for 90-day ICU all-cause mortality), indicating a change in the mortality risk relationship, which is negatively correlated below these thresholds and positively correlated above them. Fourth, subgroup analysis revealed a significant interaction between age and obesity with ABE quartiles. Finally, combining ABE with the SOFA score significantly improved the predictive performance for 30-day and 90-day ICU all-cause mortality.

### Mechanistic insights of ABE in sepsis prognosis

The correlation between ABE and mortality in sepsis may be explained by several mechanisms: first, sepsis is often accompanied by systemic inflammatory response syndrome, which can directly stimulate the glycolytic pathway through inflammatory mediators (such as tumor necrosis factor-α and interleukin-6), increasing lactate production. High levels of lactate indicate significant tissue hypoxia, which usually suggests severe illness and increased risk of mortality [20]. In addition, liver dysfunction in sepsis patients leads to reduced lactate clearance [9]. Second, lactate accumulation is a significant cause of metabolic acidosis, which itself has detrimental effects on multiple organ systems, including myocardial depression, reduced vascular responsiveness, immune suppression, and altered enzyme activity. These factors collectively exacerbate organ dysfunction and increase mortality in sepsis patients [20–22]. Furthermore, the interaction between inflammation and metabolic disturbances leads to elevated lactate levels and changes in acid–base excess, creating a vicious cycle that results in persistently negative ABE values, a condition often associated with higher mortality [5] In addition, sepsis patients often receive saline for fluid management, and hyperchloremic acidosis, which is common in clinical practice, can further increase negative ABE values [22]. In addition, high ABE values may indicate metabolic alkalosis due to hyperventilation, bicarbonate therapy, volume depletion, or electrolyte imbalance, all of which impair oxygen delivery and cardiovascular stability, leading to increased mortality. Moreover, hepatic dysfunction associated with sepsis may reduce ammonia clearance, further contributing to systemic alkalosis. Together, these mechanisms explain why both low and high ABE values are associated with increased mortality, showing a *U*-shaped association.

ABE is also linked to sepsis-related complications through various mechanisms. The kidneys are crucial in maintaining acid–base balance, and sepsis-related acute kidney injury can result in the buildup of acidic substances and a loss of buffering capacity [21], manifesting as negative ABE. Poor tissue perfusion and systemic inflammatory responses can exacerbate sepsis, leading to septic shock and further tissue hypoxia, creating a vicious cycle [23]. In patients presenting with acute respiratory distress syndrome, both respiratory and metabolic acidosis may be present due to disruption of the alveolar–capillary barrier, impaired oxygen exchange, and limited carbon dioxide excretion. This combined acidosis significantly decreases ABE, indicating severe metabolic derangement and a poor prognosis [24].

### Comparison with previous study

To date, only one study has been conducted on the relationship between ABE and death in patients with sepsis (*N* = 1178). Cantos et al*.* also reported a *U*-shaped relationship between ABE and hospital mortality in sepsis patients, after Inverse Probability of Treatment Weighting, compared with neutral ABE (≥ − 3 and < 4 mmol/L), negative ABE (< − 3 mmol/L) was associated with a higher risk of hospital mortality (HR = 1.56; 95% CI 1.11–2.18), while there was no significant difference in the risk of hospital mortality among patients with positive ABE (≥ 4 mmol/L) [13]. However, this study provides a more nuanced understanding of the critical effect of ABE in a cohort of more than 17,000 ICU sepsis cohort. Specifically, our threshold analysis found inflection points of 2.5 mmol/L for 30-day mortality and 2.2 mmol/L for 90-day mortality, suggesting that there are different optimal cutoffs for risk stratification. Unlike the classifications (negative, neutral, positive ABE) used in previous studies, our approach highlights a *U*-shaped relationship, where both significantly low and high ABE values are associated with increased mortality. This distinction suggests that a more refined threshold can better capture the complex interactions of metabolic disorders. These findings hold important implications for clinical practice. Identifying distinct inflection points for 30-day and 90-day mortality allows for more tailored risk stratification in critically ill sepsis patients. The discovery of a *U*-shaped relationship between ABE and mortality further underscores the complexity of metabolic disturbances, suggesting that both extremes of ABE warrant close monitoring. This nuanced understanding may facilitate the development of individualized therapeutic strategies aimed at correcting severe acid–base imbalances.

### Clinical implications and future research directions

Based on our findings, the combination of ABE and the traditional score for ICU mortality, the SOFA score, [25] plays a key role in improving the accuracy of mortality prediction in sepsis patients. This combined method provides a more reliable assessment of sepsis prognosis, highlighting the added value of ABE in improving the performance of the traditional SOFA model. A significantly negative ABE accurately reflects metabolic disturbances and helps in the early identification of sepsis patients at high risk due to metabolic imbalances. Changes in ABE, whether improvement or deterioration—can directly reflect shifts in the patient’s metabolic status, indicating whether the treatment is effective or the condition is worsening. ABE is derived from routine blood gas analysis data and can be obtained through simple calculations, making it widely applicable to various types of sepsis patients. However, ABE’s sensitivity to fluctuations in lactate levels—affected by liver function, systemic inflammation, and clinical interventions (e.g., fluid resuscitation and vasopressor agents), [26, 27] which may introduce variability in its predictive accuracy, particularly in specific clinical contexts.

Specifically, the observed association between high ABE levels and worse outcomes highlights the complex physiological implications of its components, particularly BE and lactate. Our analysis demonstrated that elevated ABE values were primarily driven by markedly positive BE, while lactate remained elevated but did not appear to be the primary driver, indicating a predominantly metabolic alkalosis with a potential contribution from lactic acidosis. A large BE typically reflects metabolic alkalosis, which may arise from conditions, such as hypochloremia, hypokalemia, prolonged vomiting, or excessive bicarbonate therapy [28]. These disturbances can impair oxygen delivery at the tissue level through a leftward shift in the oxygen–hemoglobin dissociation curve, exacerbate systemic hypoperfusion, and worsen hemodynamic instability [29]. Concurrently, elevated lactate serves as a marker of ongoing tissue hypoxia and cellular stress, reflecting a state of metabolic derangement rather than recovery [30]. The coexistence of these abnormalities likely represents a maladaptive response to systemic stress, contributing to microvascular dysfunction, impaired organ perfusion, and multi-organ failure, which could explain the increased mortality associated with high ABE levels. These findings underscore the importance of interpreting high ABE values in conjunction with its individual components and the broader clinical context, rather than as an isolated biomarker. Further research is warranted to elucidate these mechanisms and to determine whether metabolic disturbances identified by ABE could become targets for intervention or remain primarily useful for clinical monitoring.

ABE enhances acid–base assessment by integrating both lactate-dependent and lactate-independent disturbances, making it valuable in ICU settings. It helps differentiate metabolic acidosis causes, distinguishing lactate-driven hypoxia from non-lactate imbalances, such as renal dysfunction or hyperchloremic acidosis, guiding tailored resuscitation. ABE is also more stable in patients receiving fluid resuscitation or mechanical ventilation, where BE may be confounded by hyperchloremic acidosis or respiratory alkalosis. Clinically, ABE is easily implemented as it is derived from lactate and BE of standard arterial blood gas analysis, requiring no additional testing, making it a practical and cost-effective tool for risk stratification in sepsis patients.

According to the current evidence, a significantly negative ABE value is consistently related to increased mortality, positioning ABE as an important biomarker for adverse prognosis of sepsis patients. The simplicity and reliability of ABE in assessing the patient’s metabolic status make it an essential component in developing robust multivariate prediction models. In addition, machine learning models trained on comprehensive data sets including ABE can identify complex nonlinear interactions and provide a more nuanced understanding of patient’s prognosis [31]. Integrating ABE into machine learning algorithms may bring significant benefits and improve the accuracy of sepsis risk stratification. This approach has the potential to improve predictive capabilities, promote early intervention, support dynamic patient monitoring, and ultimately improve the prognosis of sepsis patients.

Further studies are needed to explore ABE’s utility in different sepsis subgroups, such as pneumonia-related sepsis, abdominal infections, and sepsis in immunocompromised patients. Moreover, combining ABE with other biomarkers, such as *C*-reactive protein, interleukin-6, *D*-dimer, and fibrinogen could further improve the predictive performance. Large-scale prospective studies are required to refine the sepsis prognosis model and support its use in developing personalized treatment strategies for high-risk sepsis population in ICU setting. In addition, our results demonstrated that combining ABE with the SOFA score resulted in a statistically significant improvement in predictive performance, as measured by the AUC. However, it is important to acknowledge that this improvement, while statistically significant, may have limited clinical relevance in its current form. The modest increase in AUC highlights the need to explore further combinations of ABE with other clinical variables beyond the SOFA score. Such efforts could potentially yield novel evaluation tools with greater predictive accuracy and clinical utility. Moreover, the findings underscore the potential role of ABE as a complementary biomarker in sepsis prognosis, which warrants further investigation in larger, prospective studies. Future research should also focus on determining whether these enhancements in predictive performance translate into improved clinical decision-making and sepsis patient outcomes.

### Limitations

However, there are several limitations in this study. First, this study is a single-center retrospective analysis, which may restrict the generalizability of our findings, especially when applied to broader populations outside of ICU setting. Second, certain confounding variables that could influence outcomes, such as chronic comorbidities and medication histories, were not fully accounted for in our analysis. This may have introduced residual confounding and limited the accuracy of our findings. Third, we primarily analyzed baseline ABE values on first day after ICU admission, without considering dynamic changes in ABE levels over time, which could provide additional prognostic insights. Fourth, the use of electronic health records may have led to misclassification or incomplete recording of clinical data, thus potentially affecting the reliability of our findings. Fifth, as ABE may be dynamic and multiple consecutive ABE measurements may provide additional prognostic insights, future studies incorporating dynamic trends in ABE are warranted. Sixth, although the predictive properties of ABE were statistically significant when used in combination with the SOFA score, the absolute increase in AUC was modest, and overall AUC values remained relatively low. Future studies should explore the potential value of continuous ABE measurements and their integration with other biomarkers to enhance risk stratification in patients with severe sepsis. Finally, the ABE is highly sensitive to lactate levels, which may be affected by factors unrelated to sepsis, complicating its interpretation in different clinical scenarios. Future prospective studies should aim to validate our findings through multi-centre prospective studies.

## Conclusion

There were *U*-shaped relationships between ABE and 30-day and 90-day ICU all-cause mortality, with key inflection points at 2.5 mmol/L (30 days) and 2.2 mmol/L (90 days). These findings suggest that ABE reflects critical metabolic balance in sepsis; however, it remains unclear whether specific ABE thresholds should serve as clinical targets for intervention or primarily as biomarkers for monitoring disease progression. Further research is needed to clarify whether actively modulating ABE levels can improve patient outcomes or whether its value is best realized as a prognostic and monitoring tool. In addition, ABE demonstrates potential as a prognostic biomarker for risk stratification in critically ill septic patients, showing superior predictive performance compared to base excess and lactate. While ABE modestly improves the predictive performance of the SOFA score, its role as a complementary marker in clinical decision-making warrants further investigation.

## Supplementary Information

Additional file 1.

## Data Availability

Data will be made available on reasonable request.

## Abbreviations

ABE: Alactic base excess
ICU: Intensive care unit
SOFA: Sequential organ failure assessment
BE: Base excess
HR: Hazard ratio
CI: Confidence interval
AUC: Area under the curve
NRI: Net reclassification improvement
IDI: Integrated discrimination improvement
RCS: Restricted cubic spline
MIMIC-IV: Medical information mart for intensive care IV
IQR: Interquartile range
