# Supplementary material for: Association between alactic base excess on mortality in sepsis patients: a retrospective observational study
Source: J Intensive Care. 2025 Apr 11;13:20. doi: 10.1186/s40560-025-00789-9 (PMC11987327; doi:10.1186/s40560-025-00789-9)
Supplement: Supplementary file 1 — Additional file 1. [file 40560_2025_789_MOESM1_ESM.docx]

Supplementary Table S1. Proportion of missing values for the variable.

| Variables | n (%) |
| --- | --- |
| Height | 1709 (9.99) |
| Weight | 373 (2.18) |
| Heart rate | 106 (0.62) |
| Systolic blood pressure | 184 (1.08) |
| Diastolic blood pressure | 248 (1.45) |
| Respiratory rate | 188 (1.10) |
| Oxygen saturation | 242 (1.42) |
| Glucose | 484 (2.83) |
| Anion gap | 328 (1.92) |
| Bicarbonate | 268 (1.57) |
| Blood urea nitrogen | 425 (2.49) |
| Calcium | 2409 (14.09) |
| Chlorine | 227 (1.33) |
| Estimated glomerular filtration rate | 248 (1.45) |
| Sodium | 323 (1.89) |
| Potassium | 260 (1.52) |
| Phosphorus | 2444 (14.29) |
| Partial pressure of carbon dioxide | 1204 (7.04) |
| Potential of hydrogen | 1084 (6.34) |

Supplementary Table S2. Comparison of predictive performance among ABE, BE, and lactate for mortality prediction.

|  | AUC | 95% CI | P of DeLong test |
| --- | --- | --- | --- |
| 30-day ICU all-cause mortality |  |  |  |
| ABE | 0.580 | 0.569-0.590 | *Reference* |
| BE | 0.421 | 0.410-0.433 | < 0.001 |
| Lactate | 0.444 | 0.433-0.456 | < 0.001 |
|  |  |  |  |
| 90-day ICU all-cause mortality |  |  |  |
| ABE | 0.562 | 0.552-0.571 | *Reference* |
| BE | 0.435 | 0.424-0.445 | < 0.001 |
| Lactate | 0.454 | 0.443-0.464 | < 0.001 |

Abbreviations: ABE, alactic base excess; AUC, area under curve; BE, base excess; CI, confidence interval; ICU, intensive care unit.

Supplementary Figure S1. Heatmap of the correlation matrix for variables included in adjusted multivariable Cox proportional hazard model. ABE, alactic base excess; AFRA, acute respiratory failure; BMI, body mass index; BUN, blood urea nitrogen; CHF, congestive heart failure ; CPD, chronic pulmonary disease; CVA, cerebrovascular disease; DBP, diastolic blood pressure; eGFR, estimated glomerular filtration rate; PaCO2, partial pressure of carbon dioxide; PVD, peripheral vascular disease; RRT, renal replacement therapy; SAE, sepsis associated encephalopathy; SBP, systolic blood pressure; SOFA, Sequential Organ Failure Assessment.

Supplementary Figure S2. Variance inflation factor analysis to assess multicollinearity for variables included in adjusted multivariable Cox proportional hazard model. ABE, alactic base excess; AFRA, acute respiratory failure; BMI, body mass index; BUN, blood urea nitrogen; CHF, congestive heart failure ; CPD, chronic pulmonary disease; CVA, cerebrovascular disease; DBP, diastolic blood pressure; eGFR, estimated glomerular filtration rate; PaCO2, partial pressure of carbon dioxide; PVD, peripheral vascular disease; RRT, renal replacement therapy; SAE, sepsis associated encephalopathy; SBP, systolic blood pressure; SOFA, Sequential Organ Failure Assessment.
